# Shadow Puppets and Neglected Diseases: Evaluating a Health Promotion Performance in Rural Indonesia

**DOI:** 10.3390/ijerph15092050

**Published:** 2018-09-19

**Authors:** Johanna Kurscheid, Dan Bendrups, Joko Susilo, Courtney Williams, Salvador Amaral, Budi Laksono, Donald E. Stewart, Darren J. Gray

**Affiliations:** 1 Department of Global Health, Research School of Population Health, Australian National University, Canberra, ACT 2601, Australia; salvadorcoro@yahoo.com (S.A.); Darren.gray@anu.edu.au (D.J.G.); 2 Graduate Research School, La Trobe University, Melbourne, VIC 3083, Australia; D.Bendrups@latrobe.edu.au; 3 Music Department, Theatre and Performing Arts, Otago University, Dunedin 9016, New Zealand; dhalang@yahoo.com; 4 Queensland Conservatorium Research Centre, Griffith University, South Brisbane, QLD 4101, Australia; cewilliams13@gmail.com (C.W.); donald.stewart@griffith.edu.au (D.E.S.); 5 Yayasan Wahana Bakti Sejatera Foundation (YWBS), Semarang 50183, Indonesia; dokterbudilaksono@gmail.com; 6 School of Medicine, Griffith Health, Griffith University, South Brisbane, QLD 4101, Australia

**Keywords:** soil-transmitted helminths, health promotion, shadow puppetry, knowledge and behaviours, Indonesia

## Abstract

‘Rama and the Worm’ is a shadow puppet production targeting neglected diseases in Central Java. It is an entertainment-based intervention study to promote health by reducing the impact of parasitic diseases such as soil-transmitted helminths (STH). The study uses traditional Javanese shadow puppetry (*wayang kulit*) as a vehicle in village communities to disseminate health messages and promote behaviour change to prevent diseases caused, primarily, by inadequate sanitation and poor hygiene. The health education messages contained in the play, although using traditional characters and themes, required the creation of a completely new narrative script, using characters and plot lines familiar to the *wayang kulit* repertoire, but placing them in new situations that relate specifically to health promotion objectives. The intervention was piloted in a village in Central Java, Indonesia using a pre/post design with both qualitative and quantitative analysis. A total of 96 male and female villagers, aged between 7 and 87 years, provided both baseline and follow up data. Participant knowledge and behaviours related to gastrointestinal and helminth-related disease were assessed before and after the intervention through a questionnaire administered by interview. Results revealed statistically significant improvements in both knowledge (48.6% pre-intervention score vs. 62.8% post-intervention score, *p* < 0.001) and behaviour (77.4% vs. 80.6%, *p* = 0.004) related to gastrointestinal and helminth disease. Findings of the study indicate the *wayang kulit* performance is an effective health education tool. The results provide proof of concept with scaling up the next step forward. The *wayang kulit* production provides a significant additional component for an integrated, comprehensive approach to reduction and elimination of STH infection.

## 1. Introduction

Neglected tropical diseases (NTDs) affect more than one billion of the world’s population and cause significant impacts on the economies of the developing and under-developed countries due to loss of productivity in the workforce and high health costs. The most common of the NTDs are a group of intestinal parasitic nematodes, collectively known as soil-transmitted helminths (STH), generally referring to roundworms (*Ascaris lumbricoides*), whipworms (*Trichuris trichiura*) and hookworms (*Necator americanus* and *Ancyclostoma duodenale*). It is estimated that nearly 1 in 5 people are infected with STH globally [1] with the highest concentration in Asia, where approximately one quarter of the population is believed to host at least one species [2]. STH are typically found in tropical and sub-tropical regions, particularly where poverty is rife such as rural communities with limited access to clean water, inadequate sanitation and poor hygiene behaviour [3]. Infection occurs primarily through ingestion of parasite eggs or skin contact with motile larvae in contaminated soil [4]. Contaminated vegetables or water can also act as media for parasite transfer [5]. While mortality is rare, chronic or high intensity infections can result in decreased stamina and work output and complications during pregnancy [6,7]. In children, who are highly vulnerable to infection clinical manifestations such as malnutrition, wasting, stunting and poor cognitive function can be particularly detrimental [8,9,10].

Soil-transmitted helminths have been reported across the Indonesian archipelago with an average prevalence of 31.8% during the period 2002 to 2009 [11]. A recent systematic review of STH in South Asia and South East Asia reported a countrywide prevalence for Indonesia of 22% for *Ascaris*, 20% for hookworm and 12% for *Trichuris* [3]. Poverty permeates many Indonesian rural communities where education levels are generally low, access to clean water and adequate sanitation is limited, most households lack latrines and open defecation is common [12,13]. Characteristics such as these in combination with ideal climatic conditions, facilitate the ongoing transmission of STH.

Currently, the global STH control strategy is through periodic mass drug administration (MDA) of anthelminthic drugs (albendazole or mebendazole) to high risk groups, especially school-aged children. Whilst chemotherapy is effective at reducing worm burden and treating symptoms, efficacy varies across STH species and most importantly, it does not prevent reinfection which can occur almost immediately post-treatment [4,14,15]. Transmission interruption requires changes to hygiene behaviour, access to safe water and improvements to sanitation. In low resource settings, improving basic sanitation and access to clean water is challenging and therefore improving knowledge and awareness of STH and changing hygiene behaviour is a critical and generally low-cost option to support MDA programmes. Hence, the importance of incorporating health education into control and prevention programmes cannot be understated.

‘Entertainment-education’ (E-E) or ‘edutainment’ is the process of delivering an educational media message that is designed to be entertaining and engaging, without invoking resistance, with the purpose of increasing audiences’ knowledge, creating favourable attitudes and changing undesirable behaviours related to a particular issue [16,17]. Ensuring that the message is communicated and delivered in a culturally appropriate and acceptable manner is equally as important to maximise the effectiveness of the health educational tool. Edutainment style interventions have been shown to be highly effective in educating the public about significant health issues [18]. A recent and highly successful example is the ‘Magic Glasses’ hygiene education intervention. Targeting Chinese primary school children in Hunan Province, the short animated narrative cartoon, designed to educate viewers on STH infection and prevention, was shown to reduce the incidence of STH infection by 50% (OR = 0.5, 95% CI 0.35–0.7, *p* < 0.0001) in intervention schools compared with control schools [19]. 

The Magic Glasses example has provided proof of principle that edutainment style interventions can improve knowledge and influence behaviour related to STH infection, thus opening up the opportunity for new creative interventions to be developed. In the current study, we aimed to develop a culturally appropriate intervention designed to engage with a mixed age Javanese audience based on the ‘*wayang kulit*’, a traditional shadow puppetry production. The objective of this study was to pilot a *wayang kulit* based intervention as a vehicle to disseminate health education and promote behaviour change to prevent diseases caused, primarily, by inadequate sanitation and poor hygiene. 

## 2. Methods

### 2.1. Study Design 

**Figure 1:**
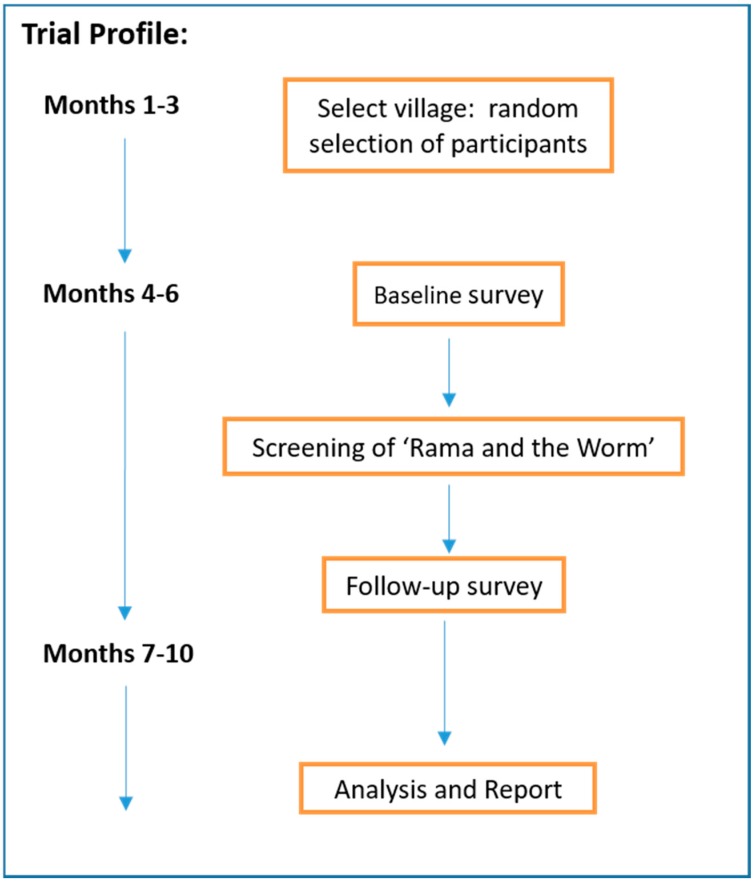
Flow chart of the Rama and the Worm study.

**Figure 2:**
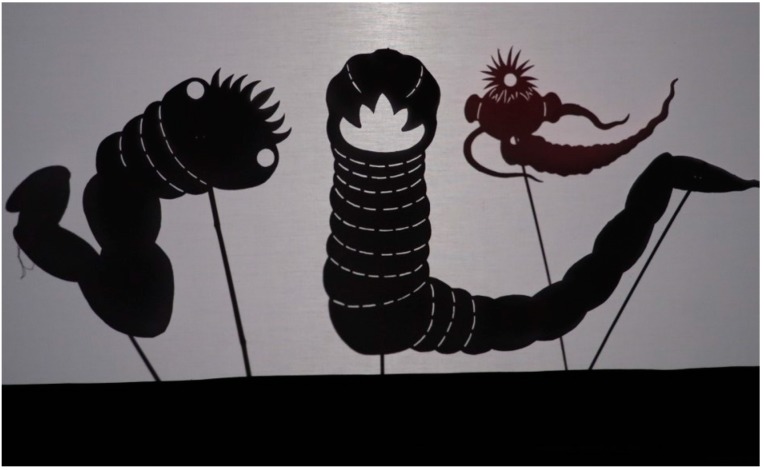
Soil-transmitted helminths represented in the ‘Rama and the Worm’ shadow puppet production.

A pre-post design (Figure 1) was used to pilot an entertainment-based health education intervention as a tool to increase awareness and improve knowledge and behaviour related to STH. In addition, this pilot study also aimed to examine the acceptability and cultural appropriateness of the intervention and to provide proof of concept. This study is part of larger study investigating the effectiveness of a squat latrine and health education package. For the current study, a traditional Javanese shadow puppetry production was developed with health education messages written into the script using characters and plot lines familiar to the *wayang kulit* repertoire. The musical accompaniment is a musical-cultural fusion involving both the traditional musical accompaniment of Javanese gamelan and Western instruments, especially rock band instrumentation. The production was filmed and presented to the participants in Bahasa Indonesia in video format. Further information on the development of the shadow puppetry, titled ‘Rama and the Worm’ (RATW) can be found in [20]. Figure 2 shows a still shot taken from RATW. An English version of the complete production can be viewed at the following: https://www.youtube.com/watch?v=AafNmmMlrvQ. The study was approved by Griffith University Human Research Ethics Committee (26 June 2017), reference No. 2016/442.

### 2.2. Study Location and Participants

The study was conducted in the village of Candi Mulyo in the district of Wonosobo, Central Java, Indonesia. Wonosobo is largely rural. It is estimated that more than half of households do not have adequate latrines and open defecation is commonly practiced [21]. The research was facilitated through the NGO Yayasan Wahana Bakti Sejatera (YWBS) Foundation. Discussions took place with local leaders and education specialists prior to the study. Their support was gained, as critical decision-makers, for the project. 

Initially the village leaders were approached, the study was explained and their support was canvassed. Following initial meetings, a comprehensive information sheet was provided to all households explaining the study and requirements for inclusion, such as agreeing to view the RATW production and being available for data collection. Interviewers then went to a random selection of 100 households, identified eligible residents (detailed below) and obtained their informed consent to participate. Only those who agreed to participate in the project in a household were interviewed using the ‘Rama HELP (Helminth Education and Latrine Project)’ questionnaire at baseline (see Supplementary Files A and B). Sample size was calculated using Medcalc [22] and using differences in proportion at 95% confidence interval and power of 80%. From prior experience in Wonosobo (8 dusuns currently involved in a larger STH prevention study) it was anticipated that about 10% of the residents interviewed would have reasonable knowledge and practices of STH at pre-intervention and that this was expected to increase to 30% post-intervention.

The sample size was 62 with a ratio of 1 between the two groups. However, it was decided to select a sample size of 100 people from the village in order to cater for loss of subjects through absence, ill health or lack of availability for follow-up, in order to maximise the number of people that complete both pre- and post- interviews.

After the baseline data collection, the ‘Rama and the Worm’ video was shown to the village. Two weeks after the video was screened the follow-up took place.

Inclusion criteria were (1) the respondent is aged 5 years of over; (2) the respondent agrees to watch the screening of ‘Rama and the Worm’; (3) the respondent is capable of understanding and responding to the questions asked by interviewers; (4) the respondent (or his/her parent) agrees to, and signs, the Informed Consent form.

### 2.3. Data Collection

Structured and semi-structured questionnaires were developed. The structured questionnaire comprised six sections eliciting details on demographic background, household characteristics, latrine use, knowledge and practices related to STH, and the presence of symptoms. The questionnaire was administered by interview in 2016 to the same cohort before and two weeks after viewing the video.

### 2.4. Data Management and Analysis

Knowledge and behaviour were assessed using a scoring system based on the number of correct responses. Knowledge scores were based on 14 questions (Supplementary Table S1) points) relating to gastrointestinal and helminth-related disease transmission, symptoms and prevention with a maximum score of 27 points. Behaviour scores were based on 17 questions (Supplementary Table S2) relating to handwashing and other hygiene practices with a maximum score of 60 points. All questions with yes or no responses were allocated a maximum of 1 point for a correct answer. Knowledge questions with scale-like responses were graded with optimal answers allocated the highest score of 2 (e.g., strongly agree) and least desirable answers (e.g., strongly disagree) with zero points. Likert type behaviour questions were similarly graded, except the most ideal responses were allocated 4 points, reducing down to zero for least ideal responses. For example, if a respondent said they “always” wash their hands before eating they would be allocated the maximum number of points (i.e., 4), “often” would receive 3 points, “sometimes” with 2 points, occasionally with 1 point and “never” with 0 points. For all questions, zero points were allocated for “don’t know” or where no response was provided. Henceforward, the term ‘score’ refers to scores as a percentage of the maximum number of points possible (87 points). Score differences were calculated by subtracting the pre-intervention score from the post-intervention score and thus reflect the percentage point difference between the two.

Statistical analyses were conducted in R version 1.1.442 (R Core Team, Vienna, Austria, 2018) and Microsoft Excel. Categorical data were analysed using Pearson’s chi-squared (χ^2^) test, respectively. Comparisons of two-level categorical and interval continuous data were analysed using the Welch Two Sample *t*-test and where categorical data consisted of more than two levels, ANOVA was used. Post-hoc analysis of ANOVA results were assessed using the Tukey HSD test. Univariate logistic regression was used to determine if demographic background could predict a positive effect from viewing the intervention, thereby allowing us to determine to whom the intervention was most effective. Univariate logistic regression analysis and odds ratios calculations using a 95% confidence interval were performed using R version 3.4.0 (R Core Team, Vienna, Austria, 2018).

## 3. Results

### 3.1. Respondent Demographics

A total of 129 people were recruited, of which 109 completed the survey at baseline. At follow up, a loss of 13.8% of respondents was reported due to not being available, leaving a total of *n* = 96 participants who completed the survey. The data presented here refer to the *n* = 96 individuals from which both baseline and follow up data were collected. The cohort consisted of roughly equal numbers of males (*n* = 47) and females (*n* = 49). Age of participants ranged between 7 and 87 (mean = 34.8 years, SD = 16.5). The majority of participants (67.7%) had an elementary level of education and more than a third (*n* = 25) worked in agriculture (farmer/plantation worker). Mean monthly household income was 1,615,441 IDR (SD = 2,149,406), equivalent to approximately 120 USD. Further demographic details are provided in Supplementary Table S1.

### 3.2. Participant History of Gastrointestinal Disease and Presence of Symptoms

In the three months prior to the baseline survey, 12.5% (*n* = 12) of participants had been diagnosed with worms in their stool on an average of 2.7 (range: 1–20) occasions. Treatment was sought by 10 of those diagnosed, mostly (70%) with a health professional (e.g., physician, public health centre staff or midwife). The remaining three individuals used medication (*n* = 1 with traditional medicine).

### 3.3. Latrine Habits

More than half (*n* = 50) of our study participants lived in a house without a latrine, with inadequate finances (64.0%) reported as the main limiting factor. The vast majority (72.0%) of these participants relied on public latrines whilst 10% used a neighbour or relative’s latrine and 18% openly defecated into nearby rivers or bushes. Of the (*n* = 46) participants who lived in a dwelling with a latrine, two did not utilise it and visited a public latrine instead. The latrines were not reported to be broken and no further explanation was provided. Less than 40% (*n* = 18) of the latrines located in participant’s homes were classified as improved by WHO standards (i.e., connected to a septic tank (i.e., improved) and among the unimproved latrines (*n* = 28), more than two thirds (*n* = 19) flowed into a river or fish pond and the remaining one third were pit type latrines. Most (93.5%) latrines were located indoors with floor spaces that were at least quarter to fully tiled or cemented (78.3%). With regard to cleaning practices after defecation, the preferred method was to use water in the bathroom (96.9%). Cleaning oneself in the river (2.1%) or with paper in the bathroom (1.0%) were also mentioned.

### 3.4. Knowledge of Gastrointestinal and Helminth-Related Disease Transmission, Symptoms and Prevention

**Figure 3:**
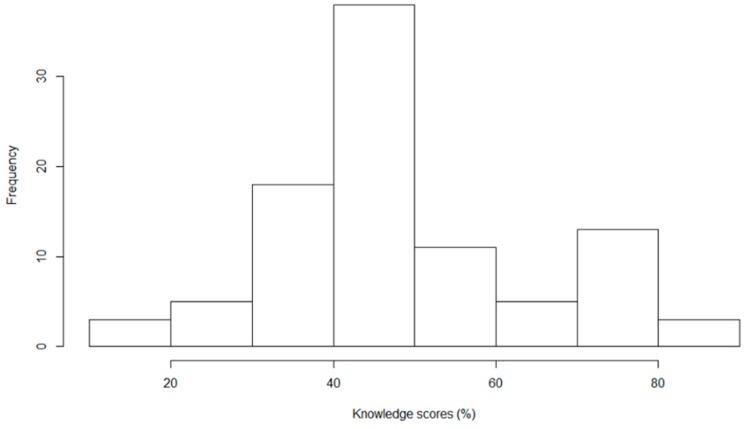
Frequency of baseline knowledge scores of the *n* = 96 study participants.

Baseline level of knowledge of gastrointestinal and helminth-related disease was limited, although there was considerable variation (Figure 3). Mean score at baseline was 48.6% (SD = 16.0). Knowledge of bowel infection causes and symptoms of round worm (*A. lumbricoides*) infection were especially poor (Table 1). Participants were also largely unaware that human faeces can contain infectious agents that can make people ill and that faeces of seemingly healthy individuals can also contain disease causing agents such as parasitic worm eggs. Knowledge of positive sanitation practices was also limited, given that less than half of the cohort was aware that defecating in the river or bush can spread diseases and is not considered good health behaviour. In contrast, nearly all (91.7%) participants knew that worms can cause illness.

**Figure 4:**
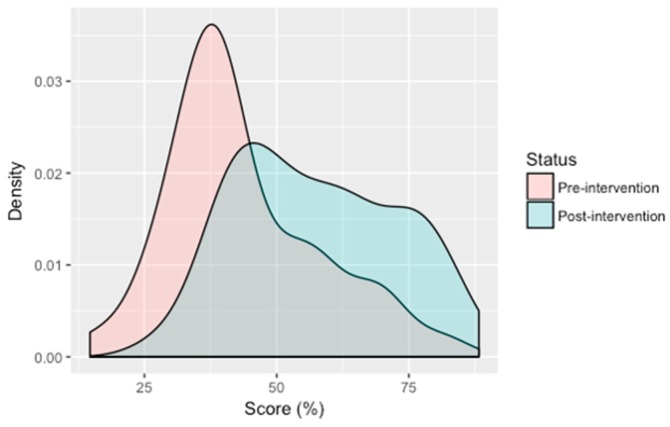
Density plot of pre- and post-intervention knowledge scores.

After the intervention, knowledge scores improved on average by 14.3 (SD = 17.2) percentage points, although 26.0% (*n* = 25) of participants showed no improvement. Nonetheless, Figure 4 reveals that overall, participant knowledge improved after the intervention. Improvements were seen across all knowledge questions assessed (Table 1) and demographic groups (Supplementary Table S3), except among unemployed participants. Relatively small gains in improvement were also observed among 7 to 12 year olds (mean increase = 5.8, SD = 11.1), 40 to 49 years (5.3, SD = 14.5), 60 years and over (7.4, SD = 9.9), students (8.6, SD = 13.3), and those in the other employment category (6.8, SD = 18.3). Despite no statistically significant differences in knowledge score changes between genders, more than one third (*n* = 17) of females failed to improve upon their score after the intervention, which is double the proportion of males with no improvement (17.0%). A high level of education was associated with a significant increase in knowledge as seen by the 37 percentage point (F [3,92] = 4.55, *p* = 0.005) increase for senior or higher educated participants compared to 11.6 (SD = 16.1), 14.6 (SD = 15.9) and 16.6 (SD = 10.9) percentage points for elementary, junior educated and uneducated participants, respectively. Post-hoc analysis revealed score differences between the senior or higher educated group and the elementary and junior educated groups were statistically significant (*p* = 0.002 and *p* = 0.027, respectively). Participants who were employed (e.g., company employee, self-employed or farm/plantation workers) also improved in their scores more so than their non-employed counterparts (Supplementary Table S3). Knowledge scores of participants living in a house with a latrine improved on average by 16.5% compared to 12.1% for those without a latrine, although the differences were not within significant levels (*p* = 0.08). Univariate logistic regression analyses were performed but revealed no meaningful associations between demographic variables and knowledge scores (data not shown).

### 3.5. Behaviours Related to Gastrointestinal and Helminth Disease

Despite the limited knowledge described above, mean behaviour scores at baseline (Table 2) indicate that study participants generally engage in preventative behaviour related to gastrointestinal and helminth disease. Consumption of raw or uncooked vegetables and buying uncovered food from street traders were however commonly practiced. Nearly two thirds (*n* = 61) either regularly or occasionally consume uncooked vegetables and 79.2% (*n* = 76) purchase uncovered food from street traders. Using utensils for eating was also not common among participants with only 40.6% (*n* = 39) reportedly engaging in this practice on a regular basis.

**Figure 5:**
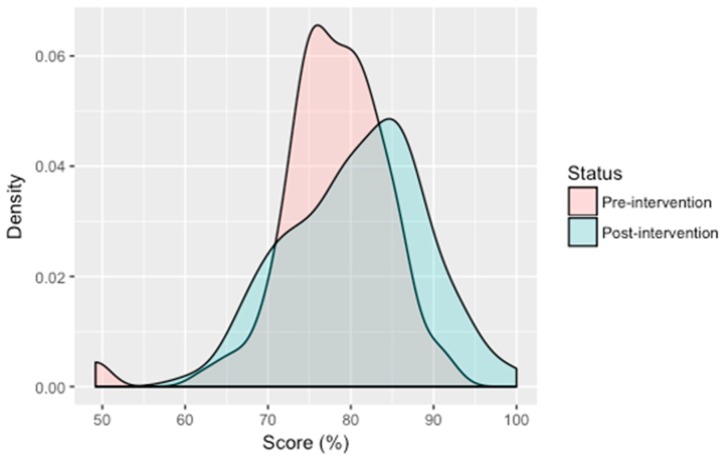
Density plot of pre- and post-intervention behaviour scores.

Behaviour scores at follow up indicate little change occurred in participant behaviour after viewing Rama and the Worm. Statistically significant improvements in score were identified for two variables: the use of utensils for eating (difference of 9.8% between pre- and post-intervention scores, *p* = 0.010) and keeping flies or other insects away from food (a difference of 7.3%, *p* = 0.048). Overall, the intervention resulted in a small yet statistically significant improvement (2.3%, *p* = 0.004), as shown in Figure 5, with a positive change in behaviour score identified in three of five participants.

## 4. Discussion

With more than 1.5 billion people estimated to be infected [1], a disability-adjusted life years (DALYs) of 3.4 million [23], and a treatment that does not prevent reinfection, STH is of major global significance. Exacerbated by climatic and socioeconomic conditions that facilitate the continual transmission of these parasites, the worst affected countries, with already overburdened economies, are struggling to combat these parasites, despite considerable efforts and resources from the global community. In Indonesia, the official control strategy for STH is annual, community-wide MDA in areas where prevalence exceeds 20% such as Central Java, the location of this study. However, local sources report that preventative chemotherapy is sporadic at best, hindered largely by a lack of resources and an inconsistent supply of albendazole and mebendazole meaning that not all provinces are covered [24] (personal communication). Even in ideal situations where all at-risk populations receive treatment, MDA programs would benefit from including a health education component aimed at increasing awareness of STH and improving hygiene behaviour to, over time, reduce environmental contamination and disease incidence.

For health information to be received positively and to be effective at reinforcing educational messages and promoting positive behaviour change among a target audience, it should be delivered in an attractive and relatable manner [16]. Furthermore, in a recent set of behaviour change communication guidelines from the Smart Development Works Organisation [25], the comment: “Within the WASH sector there is an increasing understanding that hygiene promotion requires more than business-as-usual approaches”, emphasises the need for novel and innovative programs to be created and implemented. The Magic Glasses program, trialled in China, is a prime example of the positive effect that a well-designed and executed edutainment-style health promotion initiative can have on preventing parasitic diseases [19]. Another recent example of the use of an animated film to promote health messages was a study undertaken in northern Thailand in an area where only half of the children are vaccinated against vaccine-preventable diseases, despite the government offering this service for free [26]. The edutainment intervention targeted a minority ethnic group of mothers to promote childhood vaccination. The intervention resulted in a three-fold improvement in knowledge (proportion of participants with a good knowledge at baseline = 24.6% vs. 75.4 at follow up, *p* < 0.010) and a 20% improvement in both perceptions towards childhood immunisation programmes (82.6% vs. 63.8%, *p* = 0.012) and perceived correct practices (84.1% vs. 66.7%, *p* = 0.010). The edutainment module was also positively received by both health-care providers and villagers, which is crucial for the success of any intervention.

In the current study, we describe the findings of a pilot study testing the effectiveness of a traditional Javanese shadow puppetry production (*wayang kulit*) as a vehicle to educate and promote behavioural change related to STH disease prevention in village communities in Central Java. Our findings indicate that RATW is an effective health and hygiene education tool, particularly in terms of improving knowledge of gastrointestinal and helminth-related disease. Prior to the intervention, participants knew little of what can cause bowel infections and were unfamiliar with symptoms of STH infection. Similarly, many were unaware that human faeces can contain bacteria and worm eggs, including that of visibly healthy individuals, despite the fact that nearly all participants knew that worms can cause illness. The overall low level of knowledge of gastrointestinal and helminth-related disease at baseline suggests that people living in the study area have either had limited exposure to STH- related health information or that too much time has passed since the information was received. We found that viewing RATW improved STH knowledge and awareness of approximately three quarters of our study participants, with an overall significant improvement in knowledge across all but one variable (which was already very high at baseline, i.e., 91.7%). Despite this, our findings also indicate that the intervention was more effective among some groups compared to others. This was particularly evident among higher educated participants and those who were in paid employment and although we may be inclined to assume one is associated with the other, this was not the case in our study. A 2013 study in Pahang state, Malaysia also reported better knowledge of STH and gastrointestinal-related disease among educated and employed participants compared to those who had lower education levels or were not employed [27]. The intervention also appeared to be more effective for males, given that one-third of females failed to improve their knowledge after viewing RATW, despite there being no difference in education levels. Interestingly, we also found that the intervention was less effective at improving knowledge among the very young and very old. Whether this is due to a reduced ability to interpret the messages, or a lack of interest is uncertain, as the questionnaire did not elicit information in this regard.

Although less effective at modifying behaviour than improving knowledge, the intervention succeeded in changing sanitation and hygiene related behaviour in 60% of participants. There appear to be a few behaviours that people are either reluctant or find more challenging to change, which could also signal practices common in the area that put people at greater risk of infection. Using utensils for eating and protecting food from flies or insects were the only practices that improved significantly between baseline and follow up. However, there was a small and significant improvement in overall behaviour. The generally positive heath behaviours practiced prior to the intervention may explain the relatively small change observed. It also possible that the short interval between baseline and follow up (1 month) was not long enough to induce positive modifications as behaviour change is generally a long term outcome measure [25]. Continued exposure to educational messages over a longer period of time would also likely result in a more marked improvement [28]. It is also important to highlight that due to time and financial constraints our study measured self-reported behaviour, which is known to be inherently problematic [29] given that this approach relies on participant honesty, memory and level of understanding. Therefore, hygiene practices reported here may be an over representation of true behaviour among the study population and our findings should be interpreted accordingly.

Sociodemographic background played no role in whether an individual modified their behaviour after viewing RATW, with the exception of the presence or absence of latrines in the home. Although the higher mean score difference for participants living in a home with a latrine was not found to be significantly different to that of participants without a latrine, the p-value was very close to 0.05 suggesting there may be an association that our study missed due to the small sample size. This was also the case for knowledge and latrines. Testing the intervention on a larger sample size would likely bring greater clarity as to whether a link exists between the presence of latrines in the home and knowledge improvement or behaviour change. The high level of within group variation made it difficult to determine the role of sociodemographic factors in knowledge and behaviour change in such a small sample size.

A limitation of our study was the small sample size, which made it difficult to accurately measure the variability within the study population. In addition, we did not have a control group, which would have enabled us to better assess the effectiveness. We also did not assess the effectiveness of the intervention on STH infection or compare different health educational tools. However, the main objectives of the study were to determine the cultural appropriateness and acceptability of a traditional Javanese shadow puppetry style performance and its effectiveness to disseminate health education and promote behaviour change. Given that some knowledge variables improved more than others and only a small change in behaviour was observed suggests there is room for improvement. One such example would be to test the intervention using the local Javanese dialect rather than in Bahasa Indonesia. The local dialect may have improved understanding among the audience, particularly in a rural setting such as used in this study where many of the participants had limited formal education. The findings of this study provide proof of concept enabling us to move forward and upscale into a community-based cluster-randomised intervention trial testing RATW in combination with a latrine intervention in East and West Java.

## 5. Conclusions

Whilst we acknowledge that the RATW intervention has limitations, our pilot study does indicate that with some modifications, *wayang kulit* could be an effective tool either on its own or as an addition to other more traditional approaches for improving gastrointestinal and helminth related knowledge and promoting good health behaviour. The results provide proof of concept with scaling up the next step forward. The *wayang kulit* production provides a significant additional component for an integrated, comprehensive approach to reduction and elimination of STH infection.

## Figures and Tables

**Table 1:** Comparison of participant knowledge scores pre- and post-intervention for each of the assessed questions related to knowledge of STH and gastrointestinal disease.

Knowledge Variable		Pre-Intervention Mean Score (%)	Post-Intervention Mean Score (%)	*p*-Value
Causes of bowel infections		7.3	22.7	<0.001 ^a^
Worm infection preventative measures	Wash hands before eating	50.2	77.5	0.014 ^b^
	Regularly cut fingernails	66.0	76.0	0.009 ^c^
	Wash eating/kitchen utensils with clean water	69.0	79.5	0.006 ^d^
	Keep food away from insects	65.5	79.0	0.001 ^e^
	Buy covered foods	63.5	77.0	0.001 ^f^
	Drink boiled water	70.0	79.5	0.014 ^g^
	Overall	67.1	78.2	0.001 ^h^
Worms can cause illness		91.7	95.8	0.235 ^i^
Symptoms of roundworm infection		18.7	32.7	<0.001 ^j^
Human faeces can contain bacteria and worm eggs		40.5	63.5	<0.001 ^k^
Defecating in river/bush can spread disease and worms		47.0	65.5	<0.001 ^l^
Faeces of healthy people can contain diseases and worm eggs		36.0	55.5	<0.001 ^m^
Defecating in river or garden is not good health behaviour		45.0	63.5	<0.001 ^n^
Overall knowledge score		48.6	62.8	<0.001 ^o^

^a^*t*-test: *t* = −5.25, df = 168.8, *p*-value <0.001; ^b^ *t*-test: *t* = −2.48, df = 188.6, *p*-value = 0.014; ^c^ *t*-test: *t* = −2.65, df = 189.3, *p*-value = 0.009; ^d^ *t*-test: *t* = −2.76, df = 187.8, *p*-value = 0.006; ^e^ *t*-test: *t* = −3.40, df = 187.0, *p*-value = 0.001; ^f^ *t*-test: *t* = −3.49, df = 189.4, *p*-value = 0.001; ^g^ *t*-test: *t* = −2.48, df = 186.6, *p*-value = 0.014; ^h^ *t*-test: *t* = −3.30, df = 189.1, *p*-value = 0.001; ^i^ *t*-test: *t* = −1.19, df = 173.0, *p*-value = 0.235; ^j^ *t*-test: *t* = −4.70, df = 189.5, *p*-value < 0.001; ^k^ *t*-test: *t* = −4.73, df = 180.5, *p*-value < 0.001; ^l^ *t*-test: *t* = −4.27, df = 183.7, *p*-value < 0.001; ^m^ *t*-test: *t* = −4.07, df = 183.2, *p*-value < 0.001; ^n^ *t*-test: *t* = −4.48, df = 185.5, *p*-value < 0.001; ^o^ *t*-test: *t* = −6.11, df = 189.9, *p*-value < 0.001.

**Table 2:** Behaviour scores (as % of maximum number of points) pre- and post-intervention.

Practices	Pre-Intervention Mean Score (%)	Post-Intervention Mean Score (%)	*p*-Value
Handwashing	81.0	82.1	0.439 ^a^
Soap use during handwashing	84.0	87.2	0.261 ^b^
Wearing shoes when in the paddy fields	92.2	96.2	0.132 ^c^
Wash/peel fruit	78.2	81.2	0.441 ^d^
Avoid eating raw or unboiled vegetables	56.5	63.5	0.177 ^e^
Use utensils for eating	65.0	71.2	0.010 ^f^
Avoid flies getting into food	79.5	86.7	0.048 ^g^
Avoid buying uncovered food from street vendors	48.7	52.5	0.454 ^h^
Cut fingernails frequently	92.7	96.0	0.100 ^i^
Avoid biting fingernails/sucking fingers	96.8	95.8	0.702 ^j^
Overall behaviour score	77.4	80.6	0.004 ^k^

^a^*t*-test: *t* = −0.78, df = 189.4, *p*-value = 0.439; ^b^ *t*-test: *t* = −1.13, df = 189.8, *p*-value = 0.261; ^c^ *t*-test: *t* = −1.51, df = 169.3, *p*-value = 0.132; ^d^ *t*-test: *t* = −0.77, df = 188.6, *p*-value = 0.441; ^e^ *t*-test: *t* = −1.36, df = 188.7, *p*-value = 0.177; ^f^ *t*-test: *t* = −2.61, df = 187.1, *p*-value = 0.010; ^g^ *t*-test: *t* = −1.99, df = 186.2, *p*-value = 0.048; ^h^ *t*-test: *t* = −0.75, df = 189.6, *p*-value = 0.454; ^i^ *t*-test: *t* = −1.66, df = 156.7, *p*-value = 0.100; ^j^ *t*-test: *t* = 0.38, df = 186.5, *p*-value = 0.702; ^k^ *t*-test: *t* = −2.93, df = 186.1, *p*-value = 0.004.

## Supplementary Materials

The following are available online at http://www.mdpi.com/1660-4601/15/9/2050/s1, Table S1: Knowledge questions assessed to calculate scores, Table S2: Behaviour questions assessed to calculate scores, Table S3: Association between demographic variables and knowledge and behaviour (scores presented as a percentage of maximum score). Score differences reflect the percentage point differences between baseline and follow up scores, Supplementary File A: Questionnaire (English), Supplementary File B: Questionnaire (Bahasa Indonesia).
