# Supplementary material for: Shadow Puppets and Neglected Diseases: Evaluating a Health Promotion Performance in Rural Indonesia
Source: Int J Environ Res Public Health. 2018 Sep 19;15(9):2050. doi: 10.3390/ijerph15092050 (PMC6164465; doi:10.3390/ijerph15092050)
Supplement: Supplementary file 1 [file ijerph-15-02050-s001.zip › ijerph-348676-Supplementary-File A.docx]

|  | d | d |  | m | m |  | y | y | y | y |  |  |
| --- | --- | --- | --- | --- | --- | --- | --- | --- | --- | --- | --- | --- |
| Date: |  |  | / |  |  | / | 2 | 0 | 1 | 7 | Researcher’s name |  |

**Consent checklist with agreement for watching ‘Rama and the Worm’**

| *Obtain written consent - signed letter* Y |  | N |  | ***Only proceed if ‘Yes’*** |
| --- | --- | --- | --- | --- |

***FIELD WORKERS****:* ***YOU WILL COMPLETE THIS QUESTIONNAIRE BASED ON RESPONDENTS ANSWERS. PLEASE PROMPT UNLESS OTHERWISE INDICATED.***

***THE ID and COVER SECTION MUST BE FILLED OUT FOR EVERY SINGLE PERSON INTERVIEWED.***

***INCLUSION CRITERIA CHECK***

***REMEMBER - ONLY INTERVIEW THOSE AGED 5 YEARS AND ABOVE.***

***DO NOT INTERVIEW ANYONE WHO WILL NOT BE AVAILABLE TO VIEW THE VIDEO.***

**_________________________________________________________________________**

| **RT ID** | | **House ID** | | **Individual ID** | |
| --- | --- | --- | --- | --- | --- |
|  |  |  |  |  |  |

2.6. Does **your household** have a latrine? (✓)

| 1) Yes |  | *⇒ Go to 2.8* |
| --- | --- | --- |
| 2) No |  | *⇒ Go to 2.7* |
| 3) Don't know/Refused/ No response |  | *⇒ Go to 3.1* |

2.7. (*If answer to 2.6 was* ***“No”***) why do you not have a family-latrine? (*Do not prompt* - *Multiple responses allowed*) (✓)

| 1) No money |  |  |
| --- | --- | --- |
| 2) No time to build it |  |  |
| 3) Do not need a toilet |  |  |
| 4) Other -  *Specify:* | | |
| 5) Don't Know/ Refused/ No response |  |  |
| **Skip to question 3.1** |  |  |

2.8. Where is your household latrine? (✓)

| 1) Inside the house |  |
| --- | --- |
| 2) Outside the house |  |
| 3) Don't Know /Refused/ No response |  |

2.9. Which kind of latrine is it? (✓) interviewer observe latrine?

| **Tipe of Latrine** | **Please tick √** |
| --- | --- |
| 1. WC /ST well in Septic tank |  |
| 1. WC Concrete cebluk |  |
| 1. WC Wood cebluk. |  |
| 1. WC to River/pond |  |
| 1. Broken toilet |  |
| 1. WC with damaged septic Tank |  |
| 1. WC pond |  |
| 1. No WC, use river/ gardens |  |
| 9. No WC, Use other HH toilet/public |  |

2.10. Does the latrine have cement/dry floor? (✓)

|  |  |  |  |  |  |  |
| --- | --- | --- | --- | --- | --- | --- |
| None | < 25% | 25% - 50% | 50% - 75% | >75% | 100% | Don’t know/ no response |

**4. Household demographics**

4.1. What is the Household income **per month** (Rupiah) (✓)

| 1) Enter income |  |  |  |  |  |  |  |  |  |  |  |
| --- | --- | --- | --- | --- | --- | --- | --- | --- | --- | --- | --- |
| 2) Don't know Refused/ No response |  |  |  |  |  |  |  |  |  |  |  |

4.2. Religion (✓)

| 1) Islam |  |
| --- | --- |
| 2) Other  *Specify:* |  |
| 3) Don't know Refused/ No response |  |

1. **Demographic Information**

1.1. Gender (✓)

***What is your …? (ask if not easily apparent)***

| 1) Male |  |
| --- | --- |
| 2) Female |  |

1.2. Age: (✓) **check age is >=5 to proceed.**

| 1) ENTER YEARS: |  |  |
| --- | --- | --- |
| 2) Don't Know Refused/ No response |  | |

1.3. Highest level of education completed (✓)

| 1) Elementary school |  |
| --- | --- |
| 2) Junior secondary school |  |
| 3) Senior secondary school |  |
| 4) College, or higher |  |
| 5) No school |  |
| 6) Don't know Refused/ No response |  |
| 7) At Elementary school (not completed) |  |

1.4. Employment/ job (✓)

| 1) Company (business) employee |  |
| --- | --- |
| 2) Self-employed, or entrepreneur |  |
| 3) Farmer or plantation worker |  |
| 4) Government officer/ military |  |
| 5) Home duties |  |
| 6) Student |  |
| 7) Other - Specify |  |
| 8) Not employed |  |
| 9) Don't know Refused/ No response |  |

**2. Use of latrines**

2.1. Where do you usually have a bowel motion? *Do not prompt* (✓)

| 1) River/bush |  |  |
| --- | --- | --- |
| 2) Public latrines |  |  |
| 3) Neighbours’/relatives’ latrine |  |  |
| 4) Latrine at my home |  |  |
| 5) Other  *Specify:* | | |
| 6) Don't Know Refused/ No response |  |  |

2.2. After you have a bowel motion, how do you clean yourself? *Do not prompt* (✓)

| 1) With leaves |  |  |
| --- | --- | --- |
| 2) In the river |  |  |
| 3) With paper In the bathroom |  |  |
| 4) With water in the bathroom |  |  |
| 5) Other -  *Specify:* | | |
| 6) Don't Know Refused/ No response |  |  |

**5. Helminth-related diseases**

5.1. *Over the past* ***3 months****,* ***have you been diagnosed with worms in your bowel/faeces?*** (✓) *Do not prompt*

| 1)Yes |  | How many times? |  |  | *⇒ Go to 5.2* |
| --- | --- | --- | --- | --- | --- |
| 2) No |  | *⇒ Go to 5.4* |  |  |  |
| 3) Don't know Refused/ No response |  | *⇒ Go to 5.4* |  |  |  |

5.2. (*If answer to 5.1 was “****Yes****”*) How did you treat the sickness? (*Do not prompt* - *Multiple responses allowed*) (✓) *If don't know or no response, then cannot choose in any other option.*

| 1) Bought medication at a shop |  |
| --- | --- |
| 2) Used public health care centre |  |
| 3) Went to see a physician |  |
| 4) Was hospitalised as in-patient |  |
| 5) Used traditional medicine |  |
| 6) No treatment |  |
| 7) Other *Specify:* |  |
| 8) Don't know/ do not remember/ no response |  |

5.3. (*If answer to 5.1 was “****Yes****”*) Were you absent from work or school due to worm infection? (✓)

| 1) Yes |  | How many days? |  |  |
| --- | --- | --- | --- | --- |
| 2) No |  |  |  |  |
| 3) Don't know/Refused/ No response |  |  |  |  |

5.4. In your opinion, what makes people sick with bowel infections (diarrhea, dysentery, typhoid, etc)?

(*Do not prompt but* *multiple responses allowed*) (✓)

*If don't know or no response, then cannot choose in any other option.*

| 1) Bacteria, or viruses |  |  |
| --- | --- | --- |
| 2) Worms |  |  |
| 3) Witchcraft, or Satan |  |  |
| 4) Poor diet, food, eating carelessly |  |  |
| 5) Spicy food |  |  |
| 6) Catch a cold, a cold |  |  |
| 7) Unhygienic food, water, hands, etc |  |  |
| 8) Other - *Specify:* | | |
| 9) Don't know/ Refused/ No response |  |  |

**Prevention of WORM INFECTION:**

***ASK :"Can you help prevent worm infection by.............. *insert each statement below*?"*** *Do not prompt* (✓)

|  | Strongly  disagree | Disagree | Neither  (undecided) | Agree | Strongly  agree |
| --- | --- | --- | --- | --- | --- |
| 5.5 Washing your hands before you eat? |  |  |  |  |  |
| 5.6 By regularly cutting your nails? |  |  |  |  |  |
| 5.7 Washing eating utensils or kitchen utensils with clean water (boiled water, water from sealed bottles, PAM, or artesian source)? |  |  |  |  |  |
| 5.8 Keeping food away from insects? |  |  |  |  |  |
| 5.9 Only buying foods that are covered? |  |  |  |  |  |
| 5.10 Only drinking water that was boiled? |  |  |  |  |  |

**6. WORMS**

6.2. In your opinion, can worms make you sick? *Do not prompt* (✓)

| 1) Yes |  |
| --- | --- |
| 2) No |  |
| 3) Don't know Refused/ No response |  |

6.3. What are the symptoms of Roundworm infection (Ascaris)? (*Do not prompt* - *Multiple responses allowed*) (✓) *If don't know or no response, then cannot choose in any other option.*

| 1) Fever and dizziness |  |
| --- | --- |
| 2) Coughing up phlegm (sputum) |  |
| 3) Anaemia |  |
| 4) Quickly becoming exhausted |  |
| 5) Don't know Refused/ No response |  |

6.6. Can bacteria and worm eggs be contained in human faeces? *Do not prompt* (✓)

| Strongly disagree | Disagree | Neither (undecided) | Agree | Strongly agree |
| --- | --- | --- | --- | --- |
|  |  |  |  |  |

6.7. When people pass motions in the river or bush, do you think it can spread those diseases or worms
 we mentioned above? *Do not prompt* (✓)

| Strongly  disagree | Disagree | Neither  (undecided) | Agree | Strongly  agree |
| --- | --- | --- | --- | --- |
|  |  |  |  |  |

6.8. Do you think the faeces of healthy people can also contain those diseases we

mentioned above? *Do not prompt* (✓)

| Strongly  disagree | Disagree | Neither  (undecided) | Agree | Strongly  agree | DK/refuse  No response |
| --- | --- | --- | --- | --- | --- |
|  |  |  |  |  |  |

6.9. Do you consider that passing a motion in the river or garden is good health behavior ? *Do not prompt* (✓)

| Strongly  disagree | Disagree | Neither  (undecided) | Agree | Strongly  agree | DK/refuse  No response |
| --- | --- | --- | --- | --- | --- |
|  |  |  |  |  |  |

**7. Washing hands**

***These are prompted question for when they wash hands - prompt for always, often etc***

*ASK: Do you wash your hands after the toilet all the time, often, sometimes, occasionally, or never?"* (✓)

If the participant refuses to answer any of these questions, just leave the question blank.

|  | Always  (10/10) | 2) Often  (7-9/10) | 3) Sometimes  (4-6/10) | 4) Occasionally  (1-3/10) | 5) Never  (0/10) |
| --- | --- | --- | --- | --- | --- |
| 7.1 After toilet |  |  |  |  |  |
| 7.2 Before eating |  |  |  |  |  |
| 7.3 After eating |  |  |  |  |  |
| 7.4 Before preparing food |  |  |  |  |  |
| 7.5 After changing diaper |  |  |  |  |  |
| 7.6 When coming home |  |  |  |  |  |
| 7.7 Before prayers |  |  |  |  |  |
| 7.8 Other (specify) |  |  |  |  |  |

7.9. How often do you use soap when you wash your hands? (✓)

| Always  (10/10) | 2) Often  (7-9/10) | 3) Sometimes  (4-6/10) | 4) Occasionally  (1-3/10) | 5) Never  (0/10) |  |
| --- | --- | --- | --- | --- | --- |
|  |  |  |  |  | *⇒* *If* ***“Always”***, go to 8.1 |

**8. Behaviour related to gastrointestinal diseases and worms** (✓)

Ask the question and then prompt with "always, often, sometimes, occasionally or never" - must read all options

| Question: | 1) Always (*every day*) | 2) Often (*once a week*) | 3)Sometimes (*once a month*) | 4) Occasionally | 5) Never |
| --- | --- | --- | --- | --- | --- |
| 8.1 Do you go out into the paddy fields or other fields? |  |  |  |  | If ‘Never’ Skip to 8.3 |
| 8.2 (***If answered 1)-4) above***) Do you wear shoes or sandals when you go out into the paddy fields/ other fields? |  |  |  |  |  |
| 8.3 Do you wash or peel fruit before you eat it? |  |  |  |  |  |
| 8.4 Do you eat raw or un-boiled vegetables? |  |  |  |  |  |
| 8.5 Do you eat with a spoon or a similar utensil? |  |  |  |  |  |
| 8.6 Do flies get into your food at home? |  |  |  |  |  |
| 8.7 Do you buy food from street traders if the food is uncovered? |  |  |  |  |  |

8.8. How often do you cut your fingernails? (✓)

| 1) Once in a week or more often |  |
| --- | --- |
| 2) About once in two weeks |  |
| 3) Less often than once in two weeks |  |
| 4) Don't Know Refused/ No response |  |

8.9. Do you bite or suck your fingers/ fingernails? *Do not prompt* (✓)

| 1) Yes |  |
| --- | --- |
| 2) No |  |
| 3) Don't Know Refused/ No response |  |

**9. Items checked during the visit (observations by interviewer)**

9.1. Can you show me your nails? *Do not prompt* (✓)

| 1) All clean |  |
| --- | --- |
| 2) Some dirty |  |
| 3) All dirty |  |
| 4) Refused/ No response |  |

9.2. Can you show me your hands? *Do not prompt* (✓)

| 1) Clean |  |
| --- | --- |
| 2) Somewhat dirty |  |
| 3) Very dirty |  |
| 4) Refused/ No response |  |

9.3. Do you have any itching in your anus today? (✓)

| 1) Yes |  |
| --- | --- |
| 2) No |  |
| 3) Don't Know/ Refused/ No response |  |

9.4. Are there worms in your stool today? (✓)

| 1) Yes |  |
| --- | --- |
| 2) No |  |
| 3) No bowel movement today |  |
| 4) Don't Know Refused/ No response |  |

9.5. Do you have any stomach/abdominal pain at this time? (✓)

| 1) Yes |  |
| --- | --- |
| 2) No |  |
| 3) Don't Know Refused/ No response |  |

------------------------------------------------------------------------------------------------------------------------

**Thank you very much for participating!**

| **Name of Interviewer :** |  |
| --- | --- |
| **Signature:** |  |
