# Supplementary material for: Shadow Puppets and Neglected Diseases: Evaluating a Health Promotion Performance in Rural Indonesia
Source: Int J Environ Res Public Health. 2018 Sep 19;15(9):2050. doi: 10.3390/ijerph15092050 (PMC6164465; doi:10.3390/ijerph15092050)
Supplement: Supplementary file 1 [file ijerph-15-02050-s001.zip › ijerph-348676-Supplementary-File B.docx]

|  | tg | tg |  | b | b |  | th | th | th | th |  | Nama respondent |  |
| --- | --- | --- | --- | --- | --- | --- | --- | --- | --- | --- | --- | --- | --- |
| Tanggal: |  |  | / |  |  | / | 2 | 0 | 1 | 7 |  | Tangal Lahir | -----/-----/---------- tgl/bln/thn |

**Lembar persetujuan termasuk persetujuan untuk menonton filem‘Rama and the Worm’**

| *Mendapatkan persetujuan tertulis – surat ditanda tangani* |  | T |  | Y ***Hanya dilanjutkan bila jawaban ‘Ya’*** |
| --- | --- | --- | --- | --- |

***PETUGAS LAPANGAN****:* ***ANDA AKAN MENGISI KUISIONER INI BERDASARKAN JAWABAN RESPONDEN. TOLONG TIDAK MEMBERIKAN ARAHAN JAWABAN/PENGGALIAN LEBIH JAUH KECUALI MEMANG DISEBUTKAN UNTUK MELAKUKAN HAL ITU.***

***NOMOR IDENTITAS (ID) dan HALAMAN DEPAN HARUS DIISI UNTUK SETIAP ORANG YANG DIWAWANCARAI.***

**PEMERIKSAAN KIRTERIA PENYERTAAN**

***INGAT- HANYA WAWANCARAI mereka yang berumur 5 tahun ke atas***

***JANGAN MEWAWANCARAI SIAPAPUN YANG TIDAK BERSEDIA MELIHAT VIDEO INI.***

**_____________________________________________________________________________________**

| **ID RT** | | **ID Rumah Tangga** | | **ID Perorangan** | |
| --- | --- | --- | --- | --- | --- |
|  |  |  |  |  |  |

2.6. Apakah keluarga Jenengan memiliki jamban? (✓)

| 1) Ya |  | *⇒ Lanjutkan ke 2.8* |
| --- | --- | --- |
| 2) Tidak |  | *⇒ Lanjutkan ke 2.7* |
| 3) Tidak tahu/ Menolak/ Tidak menjawab |  | *⇒ Lanjutkan ke 3.1* |

2.7. (Jika jawaban pertanyaan nomer *2.6 adalan* ***‘Tidak’***) Mengapa keluarga Jenengan tidak memiliki jamban? (*Jangan mengarahkan jawaban – Jawaban boleh lebih dari satu)* (✓)

**KEMUDIAN**: Lompat ke pertanyaan no 3.1

| 1) Tidak punya uang |  |  |
| --- | --- | --- |
| 2) Tidak punya waktu untuk membangun jamban |  |  |
| 3) Tidak terbiasa menggunakan jamban |  |  |
| 4) Lainnya  *Sebutkan:* | | |
| 5) Tidak tahu/Menolak/ Tidak menjawab |  |  |
| **Lompat ke pertanyaan no 3.1** |  |  |

2.8. Dimanakan letak jamban keluarga Jenengan? (✓)

| 1) Di dalam rumah |  |
| --- | --- |
| 2) Di luar rumah |  |
| 3) Tidak tahu/Menolak/ Tidak menjawab |  |

2.9. Apakah jenis jamban keluarga Jenengan? (✓) Petugas wawancara mengobservasi jamban?

| **Jenis Jamban** | **Please tick √** |
| --- | --- |
| 1. WC Kloset/ST baik di Septic tank |  |
| 1. WC Cebluk beton |  |
| 1. WC Cebluk kayu. |  |
| 1. WC kloset baik tp Septic ke sungai/kolam |  |
| 1. WC rusak kloset |  |
| 1. WC rusak septic Tank |  |
| 1. WC empang. |  |
| 1. Tak punya WC, BABS di Sungai,Kebun dll. |  |
| 9. Tak punya, Ikut WC KK lain/ umum |  |

2.10. Apakah jamban memiliki lantai semen/kering? (✓)

|  |  |  |  |  |  |  |
| --- | --- | --- | --- | --- | --- | --- |
| None | < 25% | 25% - 50% | 50% - 75% | >75% | 100% | Tidak tahu/Menolak/ Tidak menjawab |

**4. Kondisi demografi rumah tangga**

4.1. Berapakah pendapatan keluarga Jenengan **per bulan**? (Rupiah) (✓)

| 1) Isikan besar pendapatan |  |  |  |  |  |  |  |  |  |  |  |
| --- | --- | --- | --- | --- | --- | --- | --- | --- | --- | --- | --- |
| 2) Tidak tahu/ Tidak menjawab |  |  |  |  |  |  |  |  |  |  |  |

4.2. Agama (✓)

| 1) Islam |  |
| --- | --- |
| 2) Lainnya -  *Sebutkan:* |  |
| 3) Tidak tahu/ Menolak/ Tidak menjawab |  |

**1. Informasi Demografi**

1.1. Jenis kelamin (✓)

***Apakah jenis kelamin Jenengan …? (tanyakan jika tidak jelas terlihat)***

| 1) Laki-laki |  |
| --- | --- |
| 2) Perempuan |  |

1.2. Umur: (✓) **Periksa apakah umur >=5 untuk melanjutkan.**

| 1) MASUKKAN TAHUN: |  |  |
| --- | --- | --- |
| 2) Tidak tahu/ Menolak/ Tidak menjawab |  | |

1.3. Pendidikan tertinggi yang diselesaikan/tamat (✓)

| 1) Sekolah Dasar (SD) |  |
| --- | --- |
| 2) Sekolah Menengah Pertama (SMP) |  |
| 3) Sekolah Menengah Atas (SMA) |  |
| 4) Perguruan Tinggi atau lebih tinggi |  |
| 5) Tidak sekolah |  |
| 6) Tidak tahu /Menolak/ Tidak menjawab |  |
| 7) di sekolah dasar (Tidak tamat) |  |

1.4. Pekerjaan (✓)

| 1) Karyawan perusahaan |  |
| --- | --- |
| 2) Bekerja sendiri, or wirausaha |  |
| 3) Petani atau buruh tani |  |
| 4) PNS / tentara (ABRI) |  |
| 5) Pekerjaan di rumah tangga |  |
| 6) Siswa / Murid |  |
| 7) Lainnya - *Sebutkan:* |  |
| 8) Tidak bekerja |  |
| 9) Tidak tahu/ Menolak/ Tidak menjawab |  |

**2. Penggunaan Jamban**

2.1. Dimana biasanya Jenengan buang air besar/mbucal? Jangan mengarahkan jawaban (✓)

| 1) Sungai atau kali / semak-semak |  |  |
| --- | --- | --- |
| 2) Jamban/WC umum |  |  |
| 3) Jamban tetangga / saudara |  |  |
| 4) Jamban di rumah saya |  |  |
| 5) Lainnya -  *Sebutkan:* | | |
| 6) Tidak tahu/ Menolak/ Tidak menjawab |  |  |

2.2. Setelah buang air besar, bagaimana Jenengan membersihkan diri/cebok/cawik? *Jangan mengarahkan jawaban* (✓)

| 1) Dengan dedaunan |  |  |
| --- | --- | --- |
| 2) di sungai/kali |  |  |
| 3) Dengan tisu di kamar mandi |  |  |
| 4) Dengan air di kamar mandi |  |  |
| 5) Lainnya - *Sebutkan:* | | |
| 6) Tidak tahu/ Menolak/ Tidak menjawab |  |  |

**5. Penyakit pencernaan dan kecacingan**

5.1. *Selama* ***3 bulan*** *terakhir,* ***apakah Jenengan pernah dikatakan menderita kecacingan di pencernaan/ditemukan cacing di kotoran (tinja)?*** (✓) (Jangan mengarahkan)

| 1) Ya |  | Berapa kali? |  |  | *⇒ Lanjutkan ke 5.2* |
| --- | --- | --- | --- | --- | --- |
| 2) Tidak |  | *⇒ Lanjutkan ke 5.4* |  |  |  |
| 3) Tidak tahu/Menolak/ Tidak menjawab |  | *⇒ Lanjutkan ke 5.4* |  |  |  |

5.2. (Jika jawaban pertanyaan nomer *5.1 adalah “****Ya****”*) Bagaimana cara Jenengan mengatasi penyakit itu? (*Jangan mengarahkan jawaban – Jawaban boleh lebih dari satu*) (✓)

*Jika tidak tahu atau tidak menjawab, maka tidak boleh memilih jawaban lainnya.*

| 1) Membeli obat di apotik/toko |  |
| --- | --- |
| 2) Pergi ke Puskesmas |  |
| 3) Ke dokter |  |
| 4) Di rawat di rumah sakit / opname |  |
| 5) Menggunakan obat tradisional |  |
| 6) Tidak ada perawatan/perlakuan khusus |  |
| 7) Lainnya = *Sebutkan:* |  |
| 8) Tidak tahu/ Menolak/ Tidak menjawab |  |

5.3. (*Jika jawaban pertanyaan nomer 5.1 adalah “****Ya****”*) Apakah saat itu Jenengan tidak bisa/tidak masuk bekerja atau masuk sekolah karena penyakit perut/infeksi pencernaan? (✓)

| 1) Ya |  | Selama berapa harikah? |  |  |
| --- | --- | --- | --- | --- |
| 2) Tidak |  |  |  |  |
| 3) Tidak tahu/ Menolak/ Tidak menjawab |  |  |  |  |

5.4. Menurut pendapat Jenengan, apakah yang membuat orang menderita penyakit pencernaan/sakit perut (seperti diare, disentri, tipus dan lainnya)?

(*Jangan mengarahkan jawaban, tetapi jawaban boleh lebih dari satu*) (✓)

*Jika tidak tahu atau tidak menjawab, maka tidak boleh memilih jawaban lainnya.*

| 1) Bakteri, atau virus |  |  |
| --- | --- | --- |
| 2) Cacing |  |  |
| 3) Sihir, atau makhluk halus |  |  |
| 4) Makan tidak seimbang, makanan, makan sembarangan |  |  |
| 5) Makanan pedas |  |  |
| 6) Masuk angin, Pilek |  |  |
| 7) Makanan, air, tangan dan lainnya yang tidak bersih |  |  |
| 8) Lainnya - *Sebutkan:* | | |
| 9) Tidak tahu/ Menolak/ Tidak menjawab |  |  |

**Pencegahan kecacingan :**

***TANYAKAN :"Dapatkah Jenengan mencegah kecacingan dengan cara...............* masukkan/lanjutkan dengan setiap kalimat di bawah ini satu persatu*?"*** *Jangan mengarahkan jawaban* (✓)

|  | Sangat tidak setuju | Tidak setuju | Ragu-ragu (tidak memutuskan) | Setuju | Sangat setuju |
| --- | --- | --- | --- | --- | --- |
| 5.5. Mencuci tangan sebelum makan? |  |  |  |  |  |
| 5.6. Memotong kuku tangan secara teratur? |  |  |  |  |  |
| 5.7. Mencuci peralatan/perabotan makan dan perabotan dapur dengan air bersih (air yang didihkan, air dalam kemasan, air PAM atau air sumur bor)? |  |  |  |  |  |
| 5.8. Mencegah makanan dihinggapi atau disentuh oleh serangga(lalat)? |  |  |  |  |  |
| 5.9. Hanya membeli makanan olahan yang tertutup atau di bungkus? |  |  |  |  |  |
| 5.10. Hanya meminum air yang sudah direbus/dimasak? |  |  |  |  |  |

**6. CACING**

6.2. Menurut pendapat Jenengan apakah cacing bisa menyebabkan Jenengan sakit? *Jangan mengarahkan jawaban*

| 1) Ya |  |
| --- | --- |
| 2) Tidak |  |
| 3) Tidak tahu/ Menolak/ Tidak menjawab |  |

6.3. Apakah gejala/tanda-tanda terinfeksi/penyakit cacing gelang (Ascaris)? (*Jangan mengarahkan jawaban - Jawaban boleh lebih dari satu*) (✓) *Jika tidak tahu atau tidak menjawab, maka tidak boleh memilih jawaban lainnya.*

| 1) Demam dan pusing-pusing |  |
| --- | --- |
| 2) Batuk berdahak (mengeluarkan dahak) |  |
| 3) Kurang darah |  |
| 4) Cepat lelah |  |
| 5) Tidak tahu/ Menolak/ Tidak menjawab |  |

6.6. Apakah kotoran manusia bisa mengandung bakteri dan telur cacing? *Jangan mengarahkan jawaban* (✓)

| Sangat tidak setuju | Tidak setuju | Ragu-ragu (tidak memutuskan) | Setuju | Sangat setuju |
| --- | --- | --- | --- | --- |
|  |  |  |  |  |

6.7. Ketika orang buang air besar di sungai/kali atau di semak semak, apakah menurut Jenengan hal itu dapat menyebarkan penyakit-penyakit atau cacing yang disebutkan sebelumnya? *Jangan mengarahkan jawaban* (✓)

| Sangat tidak setuju | Tidak setuju | Ragu-ragu (tidak memutuskan) | Setuju | Sangat setuju |
| --- | --- | --- | --- | --- |
|  |  |  |  |  |

6.8. Apakah menurut Jenengan kotoran orang sehat juga dapat mengandung bibit penyakit yang disebutkan tadi? *Jangan mengarahkan jawaban* (✓)

| Sangat tidak setuju | Tidak setuju | Ragu-ragu (tidak memutuskan) | Setuju | Sangat setuju |
| --- | --- | --- | --- | --- |
|  |  |  |  |  |

6.9. Apakah menurut Jenengan membuang air besar di sungai/kali atau di kebun/semak semak adalah kebiasaan/prilaku kesehatan yang baik atau tidak? *Jangan mengarahkan jawaban* (✓)

| Sangat tidak setuju | Tidak setuju | Ragu-ragu (tidak memutuskan) | Setuju | Sangat setuju |
| --- | --- | --- | --- | --- |
|  |  |  |  |  |

**7. Mencuci tangan**

***Pertanyaan berikut adalah pertanyaan mengenai kebiasaan ketika mencuci tangan, yang diarahkan/digali lebih lanjut apakah selalu, sering dan sebagainya.***

*TANYAKAN: Apakah Jenengan mencuci tangan setelah dari jamban untuk buang air (besar atau kecil), setiap waktu/selalu, sering, sekali sekali atau tidak pernah?* (✓) (

|  | Selalu  (10/10) | Sering  (7-9/10) | Kadang-kadang  (4-6/10) | Sesekali  (1-3/10) | Tidak pernah  (0/10) |
| --- | --- | --- | --- | --- | --- |
| 7.1 Setelah dari jamban/buang air besar atau kecil |  |  |  |  |  |
| 7.2 Sebelum makan |  |  |  |  |  |
| 7.3 Setelah makan |  |  |  |  |  |
| 7.4 Sebelum menyiapkan makanan |  |  |  |  |  |
| 7.5 Setelah mengganti popok bayi |  |  |  |  |  |
| 7.6 Ketika sampai dirumah |  |  |  |  |  |
| 7.7 Sebelum sholat/sembahyang |  |  |  |  |  |
| 7.8 Lainnya (Sebutkan) |  |  |  |  |  |

7.9. Seberapa sering Jenengan memakai sabun untuk mencuci tangan? (✓)

| 1) Selalu  (10/10) | 2)Sering  (7-9/10) | 3) Kadang-kadang  (1-3/10) | 4) Jarang  (0-10) |  |
| --- | --- | --- | --- | --- |
|  |  |  |  | *⇒ Jika “Selalu”, lanjutkan ke 8.1* |

**8. Kebiasaan terkait dengan penyakit pencernaan (perut) dan cacingan** (✓)

Ajukan pertanyaannya dan kemudian arahkan/gali lebih lanjut dengan “selalu, sering, sekali-sekali, jarang, atau tidak pernah” – semua pilihan tersebut harus dibacakan

| Pertanyaan: | 1) Selalu (*setiap hari)* | 2) Sering (satu kali seminggu) | 3)Kadang-kadang | 4) Jarang | 5) Tidak pernah |
| --- | --- | --- | --- | --- | --- |
| 8.1 Apakah Jenengan pergi ke sawah atau kebun? |  |  |  |  | Lompat ke 8.3 |
| 8.2 (***Jika pertanyaan di atas terjawab 1-3***) Apakah Jenengan menggunakan sepatu atau sendal ketika ke sawah atau ke kebun lain? |  |  |  |  |  |
| 8.3 Apakah Jenengan mengupas buah buahan sebelum dimakan? |  |  |  |  |  |
| 8.4 Apakah Jenengan makan sayuran mentah/ tidak dimasak? |  |  |  |  |  |
| 8.5 Apakah Jenengan makan dengan sendok atau peralatan lainnya? |  |  |  |  |  |
| 8.6 Apakah lalat hinggap pada makanan di rumah Jenengan? |  |  |  |  |  |
| 8.7 Apakah Jenengan membeli makanan dari pedagang kaki lima jika makanannya tertutup/terbungkus? |  |  |  |  |  |

8.8. Seberapa sering Jenengan memotong kuku di jari tangan? (✓)

| 1) Sekali seminggu atau lebih sering |  |
| --- | --- |
| 2) Sekitar dua minggu sekali |  |
| 3) Lebih jarang dari dua minggu sekali |  |
| 4) Tidak tahu/ Menolak/Tidak menjawab |  |

8.9. Apakah Jenengan menggigit atau menghisap jari tangan/kuku tangan Jenengan? *Jangan mengarahkan jawaban* (✓)

| 1) Ya |  |
| --- | --- |
| 2) Tidak |  |
| 3) Tidak tahu/ Menolak/Tidak menjawab |  |

**9. Hal yang diperiksa ketika kunjungan (pengamatan oleh pewawancara)**

9.1. Bolehkan saya lihat kuku jari Jenengan? *Jangan mengarahkan jawaban* (✓)

| 1) Semua bersih |  |
| --- | --- |
| 2) Beberapa dalam keadaan kotor |  |
| 3) Semua kotor |  |
| 4) Menolak/ Tidak menjawab |  |

9.2. Bolehkan saya lihat tangan jenengan? *Jangan mengarahkan jawaban* (✓)

| 1) Bersih |  |
| --- | --- |
| 2) Sedikit kotor |  |
| 3) Sangar kotor |  |
| 4) Menolak/Tidak menjawab |  |

9.3. Apakah Jenengan merasakan gatal-gatal pada dubur (lubang pantat) hari ini? (✓)

| 1) Ya |  |
| --- | --- |
| 2) Tidak |  |
| 3) Tidak tahu/ Menolak/Tidak menjawab |  |

9.4. Apakah ada cacing dalam kotoran Jenengan hari ini? (✓)

| 1) Ya |  |
| --- | --- |
| 2) Tidak |  |
| 3) Belum buang air besar hari ini |  |
| 4) Tidak tahu /Menolak/Tidak menjawab |  |

9.5. Apakah Jenengan merasakan sakit perut saat ini? (✓)

| 1) Ya |  |
| --- | --- |
| 2) Tidak |  |
| 3) Tidak tahu/ Menolak/Tidak menjawab |  |

------------------------------------------------------------------------------------------------------------------------

**Terima kasih banyak atas partisipasinya!**

| **Nama Interviewer :** |  |
| --- | --- |
| **TTD:** |  |
