# Supplementary material for: Shadow Puppets and Neglected Diseases: Evaluating a Health Promotion Performance in Rural Indonesia
Source: Int J Environ Res Public Health. 2018 Sep 19;15(9):2050. doi: 10.3390/ijerph15092050 (PMC6164465; doi:10.3390/ijerph15092050)
Supplement: Supplementary file 1 [file ijerph-15-02050-s001.zip › ijerph-348676-supplementary.docx]

**Shadow Puppets and Neglected Diseases: Evaluating a Health Promotion Performance in Rural Indonesia**

Johanna Kurscheid, Dan Bendrups, Joko Susilo, Courtney Williams, Salvador Amaral, Budi Laksono, Donald E. Stewart and Darren J. Gray

**Table S1.** Knowledge questions assessed to calculate scores.

| **Question No.** | **Question** | **Max No. points** |
| --- | --- | --- |
| 5.4 | In your opinion, what makes people sick with bowel infections (diarrhea, dysentery, typhoid, etc)? | 3 |
| 5.5 | Can you help prevent worm infection by washing your hands before you eat? | 2 |
| 5.6 | Can you help prevent worm infection by regularly cutting your nails? | 2 |
| 5.7 | Can you help prevent worm infection by washing eating utensils or kitchen utensils with clean water? | 2 |
| 5.8 | Can you help prevent worm infection by keeping food away from insects? | 2 |
| 5.9 | Can you help prevent worm infection by only buying foods that are covered? | 2 |
| 5.10 | Can you help prevent worm infection by only drinking water that was boiled? | 2 |
| 6.2 | In your opinion, can worms make you sick? | 1 |
| 6.3 | What are the symptoms of Roundworm infection (Ascaris)? | 3 |
| 6.6 | Can bacteria and worm eggs be contained in human faeces? | 2 |
| 6.7 | When people pass motions in the river or bush, do you think it can spread those diseases or worms we mentioned above? | 2 |
| 6.8 | Do you think the faeces of healthy people can also contain those diseases we mentioned above? | 2 |
| 6.9 | Do you consider that passing a motion in the river or garden is good health behavior? | 2 |
|  | Total | 27 |

**Table S2.** Behaviour questions assessed to calculate scores.

| **Question No.** | **Question** | **Max No. points** |
| --- | --- | --- |
| 7.1 | Do you wash your hands after toilet? | 4 |
| 7.2 | Do you wash your hands before eating? | 4 |
| 7.3 | Do you wash your hands after eating? | 4 |
| 7.4 | Do you wash your hands before preparing food? | 4 |
| 7.5 | Do you wash your hands after changing diaper? | 4 |
| 7.6 | Do you wash your hands when coming home? | 4 |
| 7.7 | Do you wash your hands before prayers? | 4 |
| 7.9 | How often do you use soap when you wash your hands? | 4 |
| 8.1 & 8.2* | Do you go out into the paddy or other fields?  If yes, do you wear shoes or sandals? | 4 |
| 8.3 | Do you wash or peel fruit before you eat it? | 4 |
| 8.4 | Do you eat raw or un-boiled vegetables? | 4 |
| 8.5 | Do you eat with a spoon or similar? | 4 |
| 8.6 | Do flies get into your food at home? | 4 |
| 8.7 | Do you buy food from street traders if the food is uncovered? | 4 |
| 8.8 | How often do you cut your fingernails? | 3 |
| 8.9 | Do you bite or suck your fingernails? | 1 |
|  | Total | 60 |

* Scores were based on the combination of responses to these two questions. For example, maximum points were allocated if the respondent: never goes out into paddy fields or if they always wear shoes (regardless of frequency of visiting fields). Zero points were allocated if the respondent said they go out into fields either always or often but never wear shoes.

**Table S3.** Association between demographic variables and knowledge and behaviour (scores presented as a percentage of maximum score). Score differences reflect the percentage point differences between baseline and follow up scores.

| **Demographic variables** | ***n*** | **Knowledge score – mean (SD)** | | | | **Behaviour score – mean (SD)** | | | |
| --- | --- | --- | --- | --- | --- | --- | --- | --- | --- |
|  |  | **Baseline** | **Follow up** | **Differences** | ***p*-value** | **Baseline** | **Follow up** | **Differences** | ***p*-value** |
| Age group  7–12  13–19  20–29  30–39  40–49  50–59  60 & over | 9  12  17  22  14  16  6 | 45.3(10.3)  49.4 (17.2)  42.9 (16.0)  47.5 (16.4)  58.7 (17.7)  49.3 (16.6)  46.3 (6.9) | 51.0 (15.8)  63.9 (14.2)  63.2 (16.5)  65.0 (15.5)  64.0 (17.0)  67.8 (17.3)  53.7 (16.0) | 5.8 (11.1)  14.5 (17.1)  20.3 (22.2)  17.5 (17.9)  5.3 (14.5)  18.5 (13.9)  7.4 (9.9) | 0.090^a^ | 79.3 (4.9)  75.0 (9.7)  78.2 (9.8)  77.8 (5.7)  76.8 (6.2)  76.8 (5.4)  79.4 (4.0) | 82.6 (8.7)  78.3 (7.3)  80.4 (10.6)  83.2 (6.8)  78.4 (7.2)  78.5 (7.0)  84.4 (8.3) | 3.3 (7.6)  3.3 (9.1)  2.2 (10.6)  5.4 (7.8)  1.7 (7.5)  1.8 (8.6)  5.0 (7.9) | 0.827^b^ |
| Gender  Female  Male | 49  47 | 49.1 (17.4)  48.1 (14.6) | 62.5 (16.2)  63.3 (16.8) | 13.3 (16.4)  15.4 (18.0) | 0.557^c^ | 79.1 (5.8)  75.7 (7.7) | 81.4 (8.0)  79.7 (8.1) | 2.5 (8.8)  4.0 (7.9) | 0.378^d^ |
| Education (missing = 12)  Elementary  Junior  Senior or higher  No school | 65  16  5  10 | 49.3 (15.6)  48.6 (14.4)  34.8 (25.5)  50.4 (15.3) | 61.6 (16.5)  66.4 (13.7)  72.2 (13.2)  60.2 (19.5) | 11.6 (16.1)  14.6 (15.9)  37.0 (26.8)  16.6 (10.9) | 0.005^e^ | 78.0 (6.2)  76.1 (9.0)  72.0 (12.8)  78.3 (3.4) | 80.6 (7.7)  81.2 (8.0)  78.1 (14.1)  81.4 (7.2) | 2.5 (8.3)  3.4 (10.5)  10.0 (6.7)  3.3 (6.3) | 0.223^f^ |
| Occupation (missing = 4)  Company employee  Self-employed/entrepreneur  Farmer/plantation worker  Home duties  Student  Other  Unemployed | 4  13  25  21  18  12  3 | 50.9 (15.5)  45.9 (21.1)  43.8 (11.1)  52.0 (14.8)  48.3 (15.3)  55.2 (21.9)  46.9 (10.7) | 73.1 (12.2)  67.5 (19.4)  64.1 (16.3)  63.8 (13.9)  57.0 (15.9)  65.0 (18.5)  49.4 (8.5) | 22.2 (6.8)  21.6 (24.2)  20.3 (14.5)  11.8 (16.7)  8.6 (13.3)  6.8 (18.3)  2.5 (2.1) | 0.049^g^ | 79.2 (4.4)  75.5 (10.0)  78.1 (3.5)  79.1 (5.4)  76.8 (8.6)  75.6 (9.4)  78.3 (5.8) | 82.5 (11.3)  79.0 (10.3)  79.1 (6.5)  83.3 (7.8)  80.8 (8.0)  77.5 (6.4)  91.1 (3.8) | 3.3 (10.6)  3.5 (7.8)  1.0 (6.8)  4.2 (7.9)  4.1 (7.3)  1.9 (13.2)  12.8 (5.1) | 0.392^h^ |
| Latrine in house  Yes  No | 46  50 | 47.7 (17.8)  49.4 (14.3) | 64.2 (14.9)  61.5 (17.7) | 16.5 (20.0)  12.0 (13.7) | 0.205^i^ | 76.2 (8.2)  78.6 (5.4) | 80.7 (8.5)  80.6 (7.7) | 4.5 (8.6)  1.9 (8.2) | 0.126^j^ |

^a^ANOVA: F(6, 89) = 1.90, p = 0.090

^b^ANOVA: F(6, 89) = 0.47, p = 0.827

^c^T-test: t = 0.59, df = 92.7, p-value = 0.557

^d^T-test: t = 0.89, df = 93.0, p-value = 0.378

^e^ANOVA: F(3,92) = 4.55, p = 0.005. Post-hoc analysis revealed statistically significant differences (p<0.05) between the senior or higher education group and elementary and junior groups.

^f^ANOVA: F(3,92) = 1.49, p = 0.223

^g^ANOVA: F(6,89) = 2.21, p = 0.049. Post-hoc analysis revealed no significant differences between groups.

^h^ANOVA: F(6,89) = 1.06, p = 0.392

^i^T-test: t = 1.28, df = 83.0, p = 0.205

^j^T-test: t = 1.54, df = 93.8, p-value =0.126
